# Upregulation of annexin A1 protein expression in the intratumoral vasculature of human non–small-cell lung carcinoma and rodent tumor models

**DOI:** 10.1371/journal.pone.0234268

**Published:** 2020-06-04

**Authors:** Kevin L. Allen, Jennifer Cann, Weiguang Zhao, Norman Peterson, Michelle Lazzaro, Haihong Zhong, Herren Wu, William F. Dall’Acqua, M. Jack Borrok, Melissa M. Damschroder, Ping Tsui, Qing Li

**Affiliations:** 1 Department of Antibody Discovery & Protein Engineering, AstraZeneca plc, Gaithersburg, MD, United States of America; 2 Department of Clinical Pharmacology & Safety Sciences, AstraZeneca plc, Gaithersburg, MD, United States of America; 3 Department of Oncology, AstraZeneca, AstraZeneca plc, Gaithersburg, MD, United States of America; Sechenov First Medical University, RUSSIAN FEDERATION

## Abstract

Annexin A1 (anxA1) is an immunomodulatory protein that has been proposed as a tumor vascular target for antitumor biologic agents, yet to date the vascular expression of anxA1 in specific tumor indications has not been systematically assessed. Attempts to evaluate vascular anxA1 expression by immunohistochemistry are complicated by a lack of available antibodies that are both specific for anxA1 and bind the N-terminal–truncated form of anxA1 that has previously been identified in tumor vasculature. To study the vascular expression pattern of anxA1 in non–small-cell lung carcinoma (NSCLC), we isolated an antibody capable of binding N-terminal–truncated anxA1_27-346_ and employed it in immunohistochemical studies of human lung specimens. Lung tumor specimens evaluated with this antibody revealed vascular (endothelial) anxA1 expression in five of eight tumor samples studied, but no vascular anxA1 expression was observed in normal lung tissue. Tumor microarray analysis further demonstrated positive vascular staining for anxA1 in 30 of 80 NSCLC samples, and positive staining of neoplastic cells was observed in 54 of 80 samples. No correlation was observed between vascular and parenchymal anxA1 expression. Two rodent tumor models, B16-F10 and Py230, were determined to have upregulated anxA1 expression in the intratumoral vasculature. These data validate anxA1 as a potential vascular anti-tumor target in a subset of human lung tumors and identify rodent models which demonstrate anxA1 expression in tumor vasculature.

## Introduction

The vasculature of tumor tissue is distinct from that of normal tissue in both morphology and gene expression, and expression of multiple proteins is upregulated in tumor endothelium [1–4]. These tumor vascular markers present unique opportunities for targeting by antitumor biologics, such as ready availability to circulating drug and the potential to facilitate local accumulation of systemically administered antitumor agents [5–7]. To date, several such markers, including B7-H3, TEM8, VEGF-A/VEGFR2, PSMA, CD105, and integrin α_v_β_3_, have been explored as potential antitumor targets [8–18].

Recently it was reported that expression of the immunomodulatory protein annexin A1 (anxA1) is enhanced in tumor-associated endothelium, and an antibody targeting a membrane-associated, proteolytically cleaved form of anxA1 (anxA1_27-346_) was reported to induce rapid tumor uptake in rodent models, including models of lung cancer [19–21]. AnxA1 is known to play a role in tumor cell proliferation [22, 23] and has been shown to be involved in metastatic behavior in cancer cells, including invasion, migration, and epithelial-mesenchymal transition [24–31]. Immunohistochemistry (IHC) studies have demonstrated that anxA1 is upregulated in several tumor types, including melanoma [32], hepatocellular carcinoma [33], gastric cancer [34–36], and non–small-cell lung carcinoma (NSCLC) [37–40], and is downregulated in prostate cancer [41, 42] and many head and neck cancers [43–46]. It has been reported that the expression of anxA1 was significantly associated with the pathological grade of lung cancer while the upregulation of anxA1 correlated with decreased survival [47]. To this date, IHC analyses in these reports have focused on anxA1 expression in tumor parenchyma, and a thorough assessment of the prevalence and pattern of anxA1 expression in tumor vasculature has not been reported.

AnxA1 possesses several unique structural and functional characteristics that must be considered when studying its expression profile and function in disease states. The protein can be localized both intra- and extracellularly and exists in membrane-associated and soluble forms [30, 48, 49]. It is composed of a core domain and a unique N-terminal domain of approximately 43 residues in length. The core domain has a high degree of homology to other annexin family members and facilitates calcium-mediated binding to membranes [50]. The N-terminal domain confers many of the functional properties of anxA1 and is highly susceptible to proteolytic cleavage in a number of physiological contexts, including tumor endothelium [19, 20, 24, 51, 52]. Thus, it is particularly important to take these structural characteristics into consideration when selecting antibodies to study anxA1 expression profiles in tissue.

In this study, we generated antibodies that are specific for anxA1 and capable of binding its proteolytically cleaved form. Employing these uniquely specific antibodies, we then evaluated the expression pattern of anxA1 in human NSCLC tissue samples to determine the prevalence of vascular anxA1 expression across patient samples. We further identified rodent models of cancer that demonstrate anxA1 expression in tumor vasculature. The results presented here provide a comprehensive assessment of vascular anxA1 expression in NSCLC and address the potential of anxA1 as a vascular target for anti-tumor biologics.

## Materials and methods

### Reagents and cell cultures

AnxA1 antibody clone 686122 (catalog number MAB37701) was purchased from R&D systems (Minneapolis, MN). AnxA1 antibody clone CL0199 (catalog number AMAB90558) was purchased from Sigma-Aldrich (St. Louis, MO). AnxA1 antibody clone 29 (catalog number 610066) was purchased from BD Biosciences (San Jose, CA). AnxA1 antibody clone EPR2767(2) (catalog number ab138512) was purchased from Abcam (Cambridge, MA).

The mouse melanoma cancer cell line B16-F10-Luc2 (catalog number ATCC CRL-6475-LUC2), the Lewis lung carcinoma (LLC) cell line LL/2-Luc2 (catalog number ATCC CRL-1642-LUC2), the mouse mammary cancer cell lines 4T1-Luc2 (catalog number ATCC CRL-2539-LUC2) and Py230 (catalog number ATCC CRL-3279), and the rat mammary adenocarcinoma cell line 13762 (catalog number ATCC CRL-1666) were obtained from American Type Culture Collection (ATCC) (Rockville, MD). B16-F10-Luc2 and LL/2-Luc2 cells were cultured in Dulbecco modified Eagle medium (DMEM), and 4T1-Luc2 cells were grown in Advanced RPMI 1640 medium (Gibco, Life Technologies Europe BV, Zug, Switzerland). The 13762 cells were cultured in McCoy 5a medium, and Py230 cells were grown in F-12K Nutrient Mixture Kaighn’s Mod. (VWR, Radnor, PA) with L-glutamine, 5% HyClone FetalClone II (GE Healthcare Life Sciences, Chicago, IL), 1× Pen/Strep (Gibco) and 0.1% MITO+ serum extender (ThermoFisher Scientific, Waltham, MA).All media were supplemented with 10% fetal calf serum (Gibco). Cells were maintained in tissue culture flasks at 37°C in a humidified atmosphere with 5% CO_2_. The tumor cells were routinely subcultured two to three times per week, depending on the growth rate and split ratio. All cell lines used in the study were tested mycoplasma-free.

### Expression and purification of murine and human anxA1

Recombinant mouse and human anxA1 and anxA1_27-346_ were produced in a prokaryotic expression system. Genes encoding anxA1 antigen were digested with restriction enzymes and ligated into expression vector pET22b (Novagen) with a C-terminal 6×His tag followed by transformation into competent Top10 cells (Invitrogen) to obtain pET22-anxA1. For truncated anxA1 antigens, genes encoding 27–346 amino acids of anxA1 were cloned into the expression vector to obtain pET22-anxA1_27-346_. The anxA1 sequences were verified by DNA sequencing. Recombinant anxA1 proteins were expressed in *E*. *coli* BL21 (DE3) cells and purified with HisTrap HP columns in accordance with the manufacturer’s protocol (GE Healthcare, Piscataway, NJ). Protein concentration was measured with absorbance at 280 nm (Nanodrop; Thermo Fisher Scientific, Waltham, MA).

### Antibody generation

Human monoclonal antibodies were identified from CAT recombinant antibody phage libraries [53]. Briefly, the phage library was incubated with 2% milk and control human immunoglobulin G (IgG) to remove Fc-binding phage before binding to the anxA1-coated immunotubes (20 μg/mL in 0.1 M sodium bicarbonate buffer, pH 9.6). After a 2-hour incubation, the immunotubes were washed with phosphate-buffered saline (PBS) + 0.1% Tween. The bound phage was eluted with 1 mL of 100 mM triethylamine (Sigma-Aldrich, St. Louis, MO), neutralized with 0.5 mL of 1 M Tris HCl (pH 7.5), and used to infect log phase TG1 cells (Novagen, Darmstadt, Germany). The resulting colonies were collected and infected with helper phage M13K07 (Invitrogen, Carlsbad, CA). The infected cells were cultured overnight in 2YT media with carbenicillin (Sigma-Aldrich) and kanamycin (Sigma-Aldrich) at 30°C to generate high-titer phage.

AnxA1-binding phage was identified by enzyme-linked immunosorbent assay (ELISA), and the sequence-unique clones were converted to full-length human IgG1 as described previously [53]. For clone 4, clone 4-muIgG2a was also generated as a human-mouse chimeric antibody containing human variable regions and mouse constant regions as a tool antibody to assess human tissue in immunohistochemistry assay. The antibodies were transiently expressed in HEK293 cells, using the transfection reagent 293fectin (Thermo Fisher) according to the manufacturer’s instructions. The secreted antibody in culture supernatant was purified with a prepacked protein A column (GE Healthcare). The antibody was eluted from the column with acidic buffer (pH 3.0), neutralized, and dialyzed against PBS. The concentration of the purified antibody (molecular weight, 150 kDa) was calculated from the solution’s optical density at 280 nm.

### ELISA

ELISA plates (Costar; Corning, Corning, NY) were coated with antigen in a concentration of 2 μg/mL. After washing and blocking with 3% bovine serum albumin for 1 hour at room temperature, the phage or antibody was added to each well of the blocked plates. The plates were washed and incubated with anti–M13-HRP antibody (GE Healthcare) or antihuman Fc_γ_ (Jackson ImmunoResearch, West Grove, PA) for 1 hour before detection with SureBlue TMB peroxidase substrate (KPL, Gaithersburg, MD). The reaction was stopped with 50 μL of 0.2 M H_2_SO_4_, and the ELISA signal was read at 450 nm.

### Flow cytometry

Association of recombinant human and mouse anxA1_27-346_ to RAJI cells was induced by co-incubating cells with 2 μM protein in Tris-buffered saline (TBS) + 5 mM CaCl_2_ for 30 minutes at 4°C, followed by washing with TBS + 5 mM CaCl_2_. Cell-associated antigen binding of anti-anxA1 antibodies was measured by flow cytometry, using anti-human Fcγ labeled with Alexa Fluor 647 (Thermo Fisher Scientific) for detection. Cells were collected and distributed onto 96-well plates at 2 × 10^5^ cells per well. Cells were incubated with 50 nM antibody in fluorescence-activated cell sorting buffer (Dulbecco PBS + 5% heat-inactivated fetal bovine serum), followed by Alexa Fluor 647–labeled antihuman Fcγ secondary antibody (Jackson ImmunoResearch); each incubation was 1 hour with washes at 4°C to keep cells viable. Cells were resuspended in 1:1,000 DAPI (4′,6-diamidino-2-phenylindole) stain (Invitrogen), assayed on an LSRII flow cytometer (BD Biosciences, Franklin Lakes, NJ), and gated on a live-cell, DAPI-negative population for analysis.

### Animals

Female C57BL/6J mice approximately 5 weeks of age were obtained from Jackson Laboratories (Bar Harbor, ME). Female BALB /c (BALB/cAnNHsd) mice and female fisher rat approximately 5 weeks of age were obtained from Envigo (Indianapolis, IN). Animal were housed in individually ventilated cages on hardwood bedding and fed a commercially available diet (HarlanTeklad 2918 Diet, 18% Global Protein Diet; Harlan, Indianapolis, IN). According to the vendor’s certification program and our institutional quarterly health surveillance program, the mice were free of commonly tested rodent pathogens. All procedures were conducted in accordance with the Guide for the Care and Use of Laboratory Animals in our facility, which is accredited by the Association for Assessment and Accreditation of Laboratory Animal Care and were approved by AstraZeneca’s Institutional Animal Care and Use Committee.

### Lung metastasis tumor models

Mouse tumor cells (B16-F10-Luc2, LL/2-Luc2, 4T1-Luc2) in an exponential growth phase were harvested and centrifuged at 335 × *g* (relative centrifugal force) in a refrigerated centrifuge, and the medium was aspirated. For cell inoculation, the cell pellet was resuspended in 10× volume serum-free DMEM, filtered through a 70-μm nylon mesh cell strainer, and counted. The cell suspension was centrifuged again at 300 × *g* and resuspended in serum-free DMEM to obtain 2.5 × 10^6^ cells per mL. Mice were anaesthetized using 3% isoflurane in oxygen in an induction chamber until the respiratory rate slowed and stabilized. Each animal was then inoculated by intravenous (IV) injection in a tail vein with a single-cell suspension of more than 95% viable tumor cells in 0.2 mL of serum-free DMEM. B16-F10-Luc2 cells or LL/2-Luc2 cells were injected into C57BL/6J mice, while 4T1-Luc2 cells were injected into BALB/c mice. The total number of cells to be implanted was 0.5 × 10^6^ per mouse. Metastatic lung tumor growth in mice were monitored by bioluminescence imaging with a spectrum in vivo imaging system (PerkinElmer, Waltham, MA). To generate rat metastatic lung tumor 13762 mammary adenocarcinoma, female Fischer rats were injected via tail vein with a cell suspension of 13762 breast adenocarcinoma cells (0.5 × 10^6^ cells per rat). After inoculation, the animals were checked daily for morbidity and mortality. At the time of routine monitoring, the animals were checked for any effects of tumor growth and treatments on normal behavior, such as mobility, food and water consumption, hair matting, and other functions.

To collect lung tumors, mice were euthanized by CO_2_ inhalation. Only CO_2_ from the house supplied gas nozzle was used for euthanasia. Lung tumors were excised and fixed in 4% paraformaldehyde for 24 hours. B16-F10-Luc2 lung tumors (day 0, n = 3; day 9, n = 3; day 12, n = 3; day 15, n = 2), LLC-Luc2 lung tumors (day 18, n = 4), 4T1-Luc2 lung tumors (day 15, n = 4) and 13762 rat lung tumors (day 15, n = 5) were collected. Fixed tissues were then paraffin-embedded before IHC assessment.

### Py230 breast orthotopic tumor model (in vivo Py230 tumorigenesis)

The Py230 cell line is an epithelial-like murine mammary tumor cell line with properties similar as those of normal mouse mammary stem cells. Upon orthotopic injection in C57BL/6 mice, Py230 cells form luminal mammary tumors and lung metastases [54]. Py230 cells were harvested in exponential growth phase, washed three times with cold 1× Hank’s buffered salt solution, and filtered through a 70-μm strainer. A total of 10^6^ Py230 cells in 0.3 mL HBSS were implanted into the mammary fat pads of 6–7-week-old, lightly anesthetized C57BL/6J mice by subcutaneous injection. Following implantation, mice were monitored daily until a palpable tumor was observed. Tumor volumes were then measured by caliper three times per week and then daily once the tumor volume exceeded 1800mm^3^. Mammary tumors appeared at an average of 9 days after injection, remained approximately 200 mm^3^ in size until an average of 30 days after injection, and grew to the maximum allowed tumor size (2,000 mm^3^) by an average of 51 days after injection. Mice were euthanized when the tumor volume exceeded 2000mm^3^ or earlier if the animal(s) showed signs of distress (labored breathing, lethargy, and/or anorexia) in the study. To collect tumors, mice were euthanized by CO_2_ inhalation. Py230 tumors (day 25, n = 4; day 30, n = 4; day 35, n = 4; day 44, n = 4) were collected. Fixed tissues were paraffin-embedded before IHC assessment.

### IHC staining Human tissues

Multitype human normal organ tissue microarrays (TMAs) (Tristar Technology Group, Rockville, MD) and NSCLC TMAs (US Biomax, Derwood, MD) were used in accordance with the ethical principles of the Declaration of Helsinki and in compliance with all national and local regulatory guidelines. Tissues were sectioned at approximately 4 μm and placed on positively charged slides. After sectioning, the slides were air dried overnight before IHC staining.

To assess anxA1 expression, a chromogenic IHC assay was developed with an anti-anxA1 monoclonal antibody (clone 4-muIgG2a). Formalin-fixed, paraffin-embedded sections were deparaffinized and stained with a Discovery Ultra IHC/ISH research slide staining system (Ventana Medical Systems, Oro Valley, AZ) with a heated antigen retrieval pretreatment step (Cell Conditioner 1; Ventana). The sections were incubated with an anti-anxA1 primary antibody at a concentration of 0.6 μg/mL for 28 minutes at 36°C. The anti-anxA1 primary antibody (clone 4-muIgG2a) was detected by using secondary goat anti mouse IgG_ OmniMapHRP (catalog no. Cat #760–4310, Lot #G04502, Ventana), followed by diaminobenzidine chromogen substrate (Roche Diagnostics, Indianapolis, IN) and the slides were counterstained with hematoxylin. Nonimmune mouse isotype antibody (IgG) was included as a negative assay control.

### Mouse tissues

Briefly, 4 μm paraffin mouse lung tissue sections were mounted on glass slides and were deparaffinized. The immunostaining was performed on the Ventana Discover ULTRA autostainer (Ventana Medical Systems, Oro Valley, AZ) using OmniMapHRP detection method. The primary antibody was anxA1 antibody (clone 4-huIgG1) and was incubated with concentration at 0.5 μg/mL Following primary antibody incubation, the samples were incubated in the specific link antibody rabbit anti-human IgG at concentration 2 μg/ml (Jackson ImmunoResearch Laboratories® cat# 309-005-082) for 16 minutes. Then, primary antibody was visualized with OmniMap goat anti-rabbit HRP (catalog no. Cat #760–4311, Ventana) respectively, and DAB (catalog no. cat# 760–159, Ventana).

All IHC stained slides were assessed by an experienced board-certified pathologist (JAC). The intensity of Annexin A1 IHC staining was assessed semi-quantitatively: 0 (none), 1+ (faint) 2+ (moderate), 3+ (maximum); and the extent of staining was estimated as the percentage of tissue stained positively. In the tumor samples, neoplastic cell staining was assessed as present or absent.

### Statistical analysis

Prism software, version 8 (GraphPad), was used for data analysis. Comparison between two systems was performed by Student *t* test, and that among multiple systems was performed by one-way analysis of variance with either Dunnett test or Tukey post hoc test. A *P* value of ≤0.05 was considered to be statistically different.

## Results

### Binding characteristics of anxA1 antibodies and isolation of novel antibodies by phage panning

**Fig 1:**
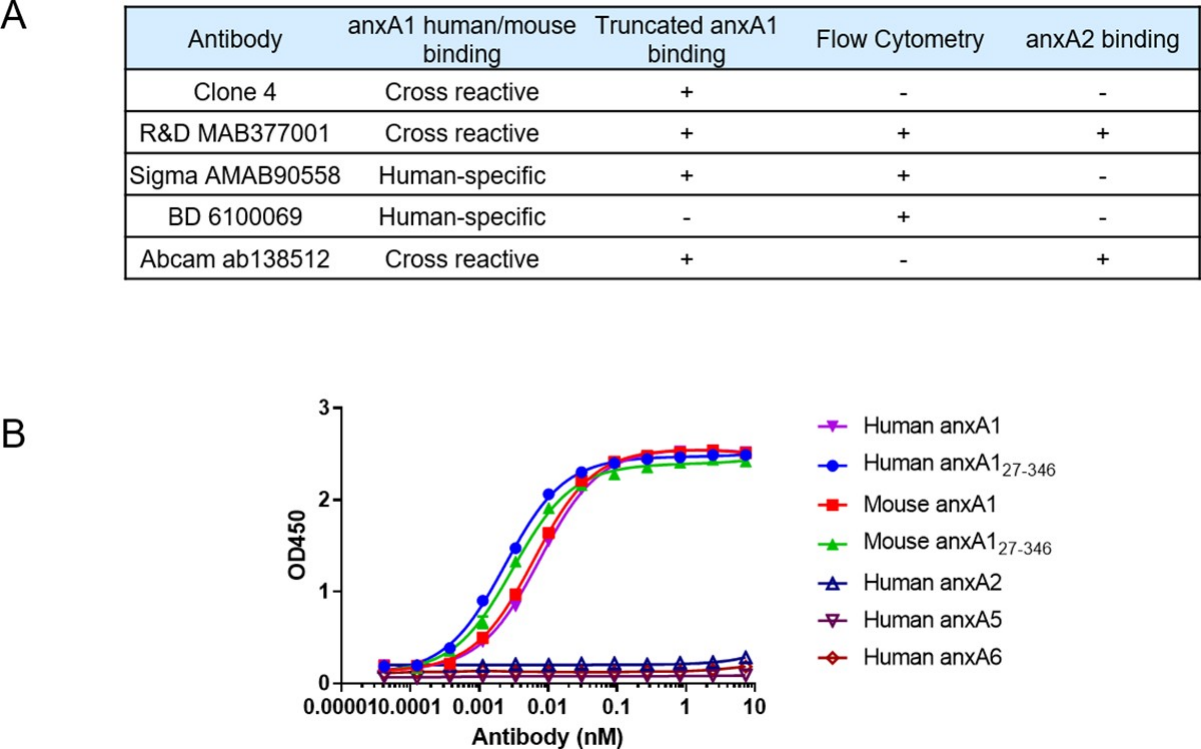
Binding characteristics of commercial anti-anxA1 antibodies. Summary of binding characteristics of commercial antibodies and clone 4-huIgG1 (A), and ELISA binding of clone 4-huIgG1 to full length and N-terminal truncated human and mouse anxA1, with negative binding to human annexin A2, A5, and A6 (B). Interpolated EC50 values: full length human anxA1 7.53 pM, full length mouse anxA1 6.19 pM, human anxA1_27-346_ 2.36 pM, mouse anxA1_27-346_ 3.09 pM based on One Site—Total Binding, least squares fit.

To characterize anxA1 expression in tumor vasculature, we initially assessed a number of commercially available anti-anxA1 monoclonal antibodies for a) binding to both human and mouse anxA1_27-346_ as previously reported in tumor vasculature, b) but not binding to other annexin family member, e.g. annexin A2 (anxA2) [19, 20]. Remarkably, none of the commercially available antibodies evaluated were found to meet the criteria of the binding capability to characterize the tumor vascular anxA1 in both human and mouse tissues. Clone CL0199 (Sigma-Aldrich) and clone 29 (BD Biosciences) did not demonstrate species cross-reactivity with mouse, and in addition, clone 29 only recognized the N-terminal domain of the protein. Clone 686122 (R&D Systems) and clone EPR2767[2] (Abcam) were not specific to anxA1 and bound to anxA2 as well (Fig 1A). Furthermore, besides characterization of anxA1 expression using IHC, we would also like to identify antibody capable of binding cell membrane–associated anxA1_27-346_ for tumor uptake imaging study in tumor vasculature anxA1 positive rodent model. Although Clone 686122 (R&D Systems), Clone CL0199 (Sigma-Aldrich) and clone 29 (BD Biosciences) were able to bind cell membrane–associated anxA1_27-346_ shown by flow cytometric assay (Fig 1A), they either have non-specific binding to anxA2 or have no species cross-reactivity with mouse. Therefore, there is a need to generate anti-anxA1 monoclonal antibodies for IHC study and in vivo tumor uptake study.

We isolated a panel of monoclonal antibodies by screening a human antibody phage library for binding to anxA1, then assessed anxA1 binding properties. From this panning campaign, we selected antibody clone 4 as a tool antibody for IHC study due to its high binding affinity (EC_50_ for human and mouse anxA1_27-346_, 2.36 pM and 3.09 pM, respectively), species cross-reactivity, and specificity for recognizing anxA1 (Fig 1B). Clone 4-muIgG2a was generated as a human-mouse chimeric antibody containing human variable regions and mouse constant regions as a tool antibody to assess human tissues in immunohistochemistry assay, while clone 4-huIgG1 as a full-length human IgG was used to assess mouse tissues. Furthermore, several additional antibodies discovered during the phage panning campaign were shown to bind cell membrane–associated anxA1_27-346_ by flow cytometry. Antibody clones 1, 77, and 84 all demonstrated binding to recombinant anxA1_27-346_ localized to the surface of RAJI cells in the presence of calcium, as well as binding by ELISA (S1 Fig).

### IHC analysis of anxA1 expression in human lung intratumoral vessels and normal tissue

**Fig 2:**
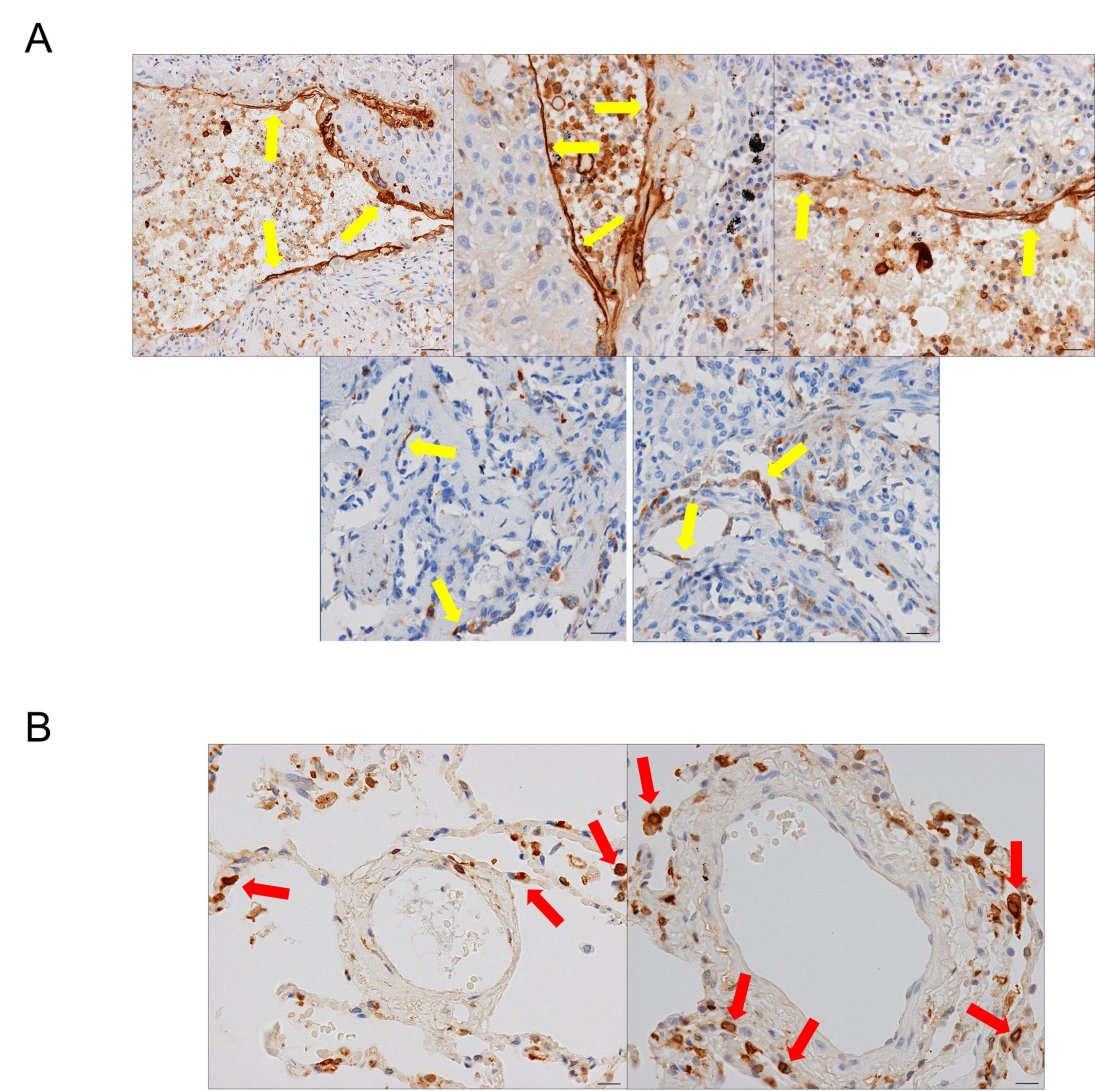
Vascular anxA1 IHC expression. Representative immunohistochemistry of human lung tumors (A) and normal lung (B) showing annexin A1 expression (brown) in the intratumoral vessels of human lung tumors, but not in the microvessels of normal lung. Vascular staining (yellow arrows) in 1–10% of total endothelial cells was observed in 5/8 human lung tumors (A), while positive staining of neoplastic cells was seen in 3/8 samples (not shown). In normal lung (B), no endothelial cell staining was observed in the 4 samples examined; however, interstitial and alveolar macrophages were strongly positive (red arrows). All scale bars = 10 μm.

Five of eight human lung tumor specimens exhibited positive staining for anxA1 in intratumoral vessels by IHC analysis (Fig 2A). The intensity of staining was 2+ (moderate) to 3+ (maximum) in the positively stained intratumoral endothelial cells. The extent of vascular endothelial staining (percent staining) for anxA1 ranged from 1% to 10% of vessel staining, with endothelium defined on the basis of architectural location and cellular morphology by an experienced pathologist. AnxA1 staining in neoplastic cells was detected in 3/8 human lung tumor specimens. There was no association between anxA1 staining in intratumoral endothelial cells and neoplastic cells. No anxA1 staining was detected in the microvessels of normal lung tissue, though anxA1 staining intensity of 1–3+ was detected in macrophages and neutrophils within all human lung tumor specimens and normal tissue specimens. The positive staining of anxA1 in macrophages and neutrophils are consistent with anxA1 expression in the immune cells as literature reported [55, 56].

AnxA1 expression in human TMAs as examined by IHC was consistent with the specimen IHC analysis. Of 80 human tumor samples, 30 exhibited 2–3+ staining for anxA1 in the intratumoral vessels (Table 1, S2 Fig). Neoplastic cell staining was observed in 54 of 80 samples. Staining was most commonly observed in the cytoplasm with or without membrane staining. No association was observed between anxA1 staining in the intratumoral endothelial cells and anxA1 staining in the neoplastic cells. In contrast, in a panel of 32 normal tissue types, vascular staining was negative in all TMAs except in kidney medulla (two of two positive) and stomach fundus (three of three positive) (Table 1).

**Table 1:** Vascular anxA1 staining in TMAs of NSCLC and normal tissues. Vascular anxA1 staining was positive in 30/80 NSCLC TMA samples, with 54/80 samples demonstrating neoplastic staining. Vascular staining in normal tissues was negative with exceptions of kidney medulla (2/2 positive vascular staining) fundus (3/3 positive vascular staining).

Organ System	Tissue type	No. of cores examined*	No. vascular positive	No. vascular negative
Respiratory	Lung	3	0	3
Immune	Lymph node	3	0	3
	Spleen	2	0	2
	Thymus	2	0	2
Cardiovascular	Heart	1	0	1
Gastrointestinal	Stomach (fundus)	3	3	0
	Stomach (muscularis)	2	0	2
	Ileum	2	0	2
	Colon (descendens)	2	0	2
	Gallbladder	2	0	2
	Liver	3	0	3
	Parotid gland	3	0	3
Integumentary	Skin	1	0	1
Reproductive	Prostate	3	0	3
	Testis	2	0	2
	Ovary	2	0	2
	Exocervix	3	0	3
	Uterus	1	0	1
	Breast	2	0	2
	Endocervix	2	0	2
	Endometrium	1	0	1
	Placenta (early)	3	0	3
	Seminal vesicle	2	0	2
	Fallopian tube	3	0	3
Urinary	Ureter	1	0	1
	Urinary bladder	1	0	1
	Kidney (cortex)	3	0	3
	Kidney (medulla)	2	2	0
Central nervous system	Cerebellum (cortex)	1	0	1
	Cerebrum	3	0	3
Endocrine	Adrenal gland	3	0	3
	Thyroid	3	0	3
Tumor	NSCLC	80	30	50

*Tissue cores were 1 mm in diameter.

### AnxA1 expression in rodent intratumoral vessel models

To identify rodent tumor models that are representative of the vascular anxA1 staining observed in human lung tumor specimens, we conducted IHC analysis of intratumoral vessels in multiple rodent models. We chose primarily metastatic and orthotopic models in this study because host immune systems in these models are not compromised, and these models better mimic tumor growth within the tissue environment than subcutaneous or xenograft tumor models [57–61]. The tumor models that were examined included a metastatic LLC model [20], a B16-F10 lung metastatic model, a 4T1 lung metastatic model, and a 13762 rat lung metastatic model [19], as well as the Py230 orthotopic breast tumor model. Luciferase-expressing LLC-Luc2 cells, B16-F10-Luc2 cells or 4T1-Luc2 cells were used in the three models for the convenience of monitoring metastatic lung tumor growth in mice by bioluminescence imaging. The tumors were harvested at the end point of the tumor growth curve.

**Fig 3:**
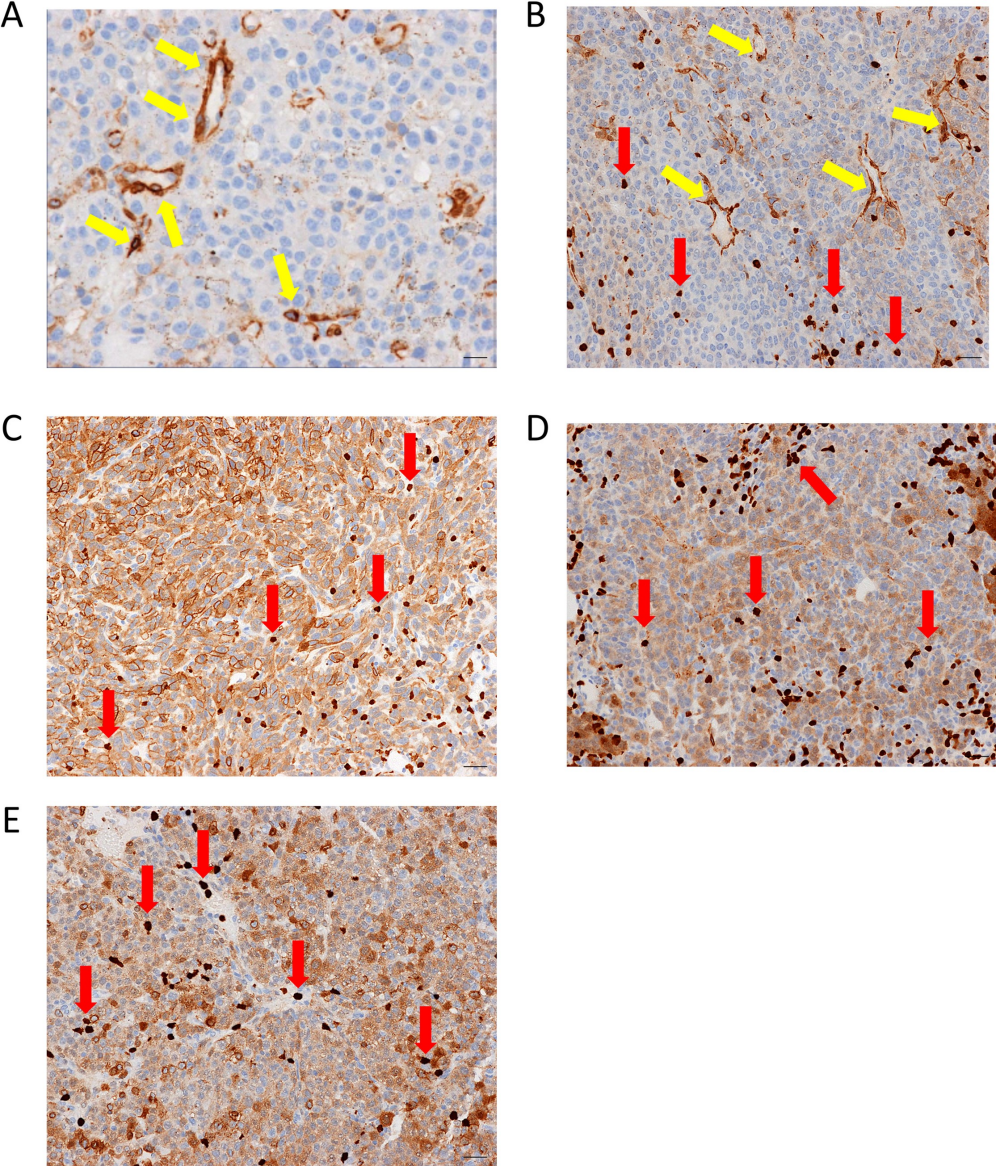
AnxA1 expression in rodent models of cancer. Representative IHC images of tumor area in (A) B16-F10-Luc2 lung tumors, (B) Py230 breast tumor, (C) LLC-Luc2 lung tumors, (D) 4T1-Luc2 lung tumors, and (E) 13762 lung tumors. AnxA1 expression (brown) was seen in endothelial cells lining microvessels (yellow arrows) in the B16-F10-Luc2 lung metastatic tumor model and the Py230 tumor model, but not in the LLC-Luc2 lung metastatic tumor model, the 4T1-Luc2 lung metastatic tumor model, or the 13762 rat lung metastatic tumor model, despite 1–3+ staining in metastatic neoplastic cells. Note that in B, C, D, and E, there is strong anxA1 expression in macrophages (red arrows). All scale bars = 10 μm.

Within a majority of B16-F10-Luc2 lung metastatic foci, a majority of neovessels were lined by endothelial cells that exhibited 2–3+ staining for anxA1in the cytoplasm, whereas very weak anxA1 staining was detected in the infiltrated and expanded neoplastic cells at 15 days after implantation (Fig 3A). Based on this finding, we subsequently tested clones 1 and 84 for tumor uptake in mice bearing B16-F10-Luc2 lung metastases that had been injected into a tail vein. Ex vivo fluorescence imaging of Alexa Fluor 680–conjugated clones 1 and 84 demonstrated no significant tumor uptake in this model as compared with isotype control (S3 Fig).

Throughout the tumor areas of the Py230 orthotopic breast tumor model, the walls of neovessels were strongly positive (2–3+) for anxA1 staining by endpoint day 44; however, the signal was localized to the vascular smooth-muscle cells, and the subjacent endothelial cells were negative for anxA1 (Fig 3B). In comparison, the small, thin-walled intratumoral neovessels were consistently negative for anxA1 staining in the LLC-Luc2 metastatic model (Fig 3C), the 4T1-Luc2 lung metastatic model (Fig 3D), and the 13762 rat lung metastatic model (Fig 3E), despite moderate to strong anxA1 staining in metastatic neoplastic epithelial cells within the three models. These results were similar to the IHC analysis of human tissue in so much as no direct association of anxA1 expression was observed between intratumoral vessel staining and neoplastic cell staining across these models.

**Fig 4:**
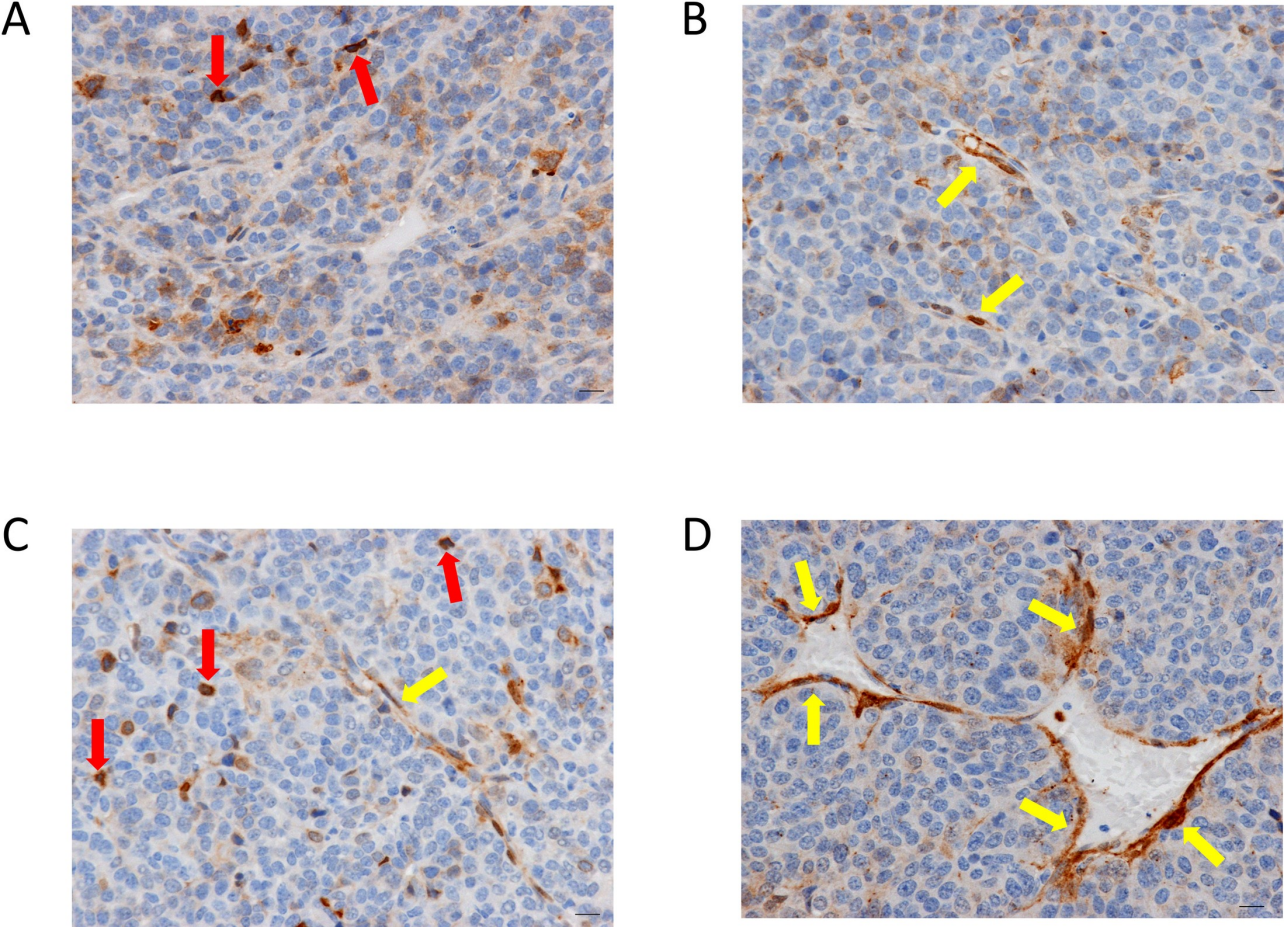
Time course of anxA1 expression in Py230 orthotopic breast tumor model. Immunohistochemical expression of anxA1 (brown) in Py230 tumors at (A) day 25, (B) day 30, (C) day 35, and (D) day 44. Microvascular endothelial cell expression of anxA1 (yellow arrows) begins at day 30 and increases in extent and intensity through day 44. Note that macrophages also exhibit strong anxA1 expression (red arrows). All scale bars = 10 μm.

To further understand the timing of anxA1 expression in intratumoral vessels, we undertook a time course IHC study in the Py230 orthotopic breast tumor and B16-F10-Luc2 lung metastatic models. In the Py230 model, anxA1 expression was exhibited in approximately 10% of tumor vessels by day 30 and increased to 50–75% by termination at day 44 (Fig 4).

**Fig 5:**
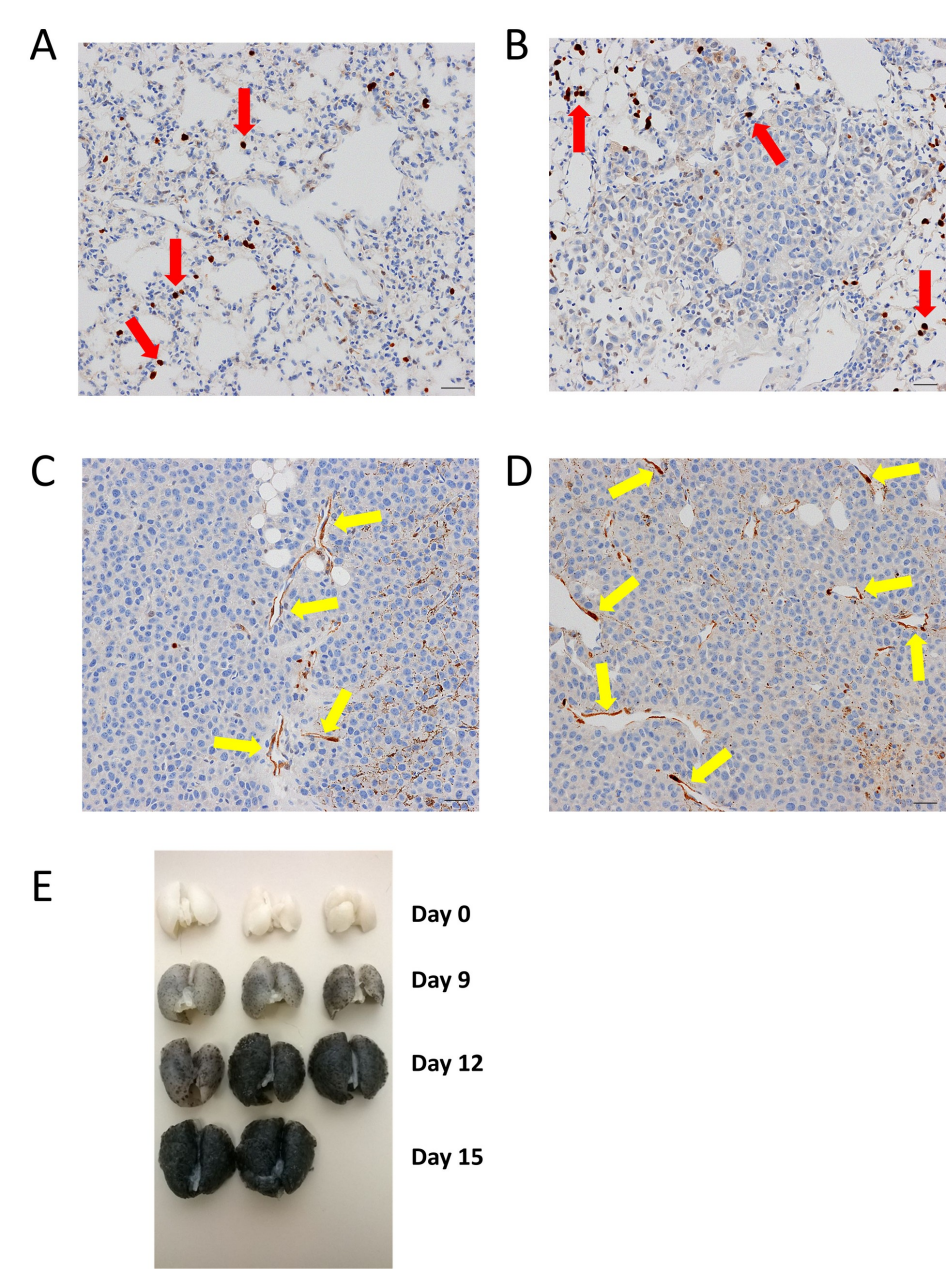
Time course of anxA1 expression in B16-F10-Luc2 lung metastases. Immunohistochemical expression of anxA1 (brown) in B16-F10-Luc2 tumor-bearing lungs at (A) day 0, (B) day 9, (C) day 12, and (D) day 15. Microvascular endothelial cell expression of anxA1 (yellow arrows) begins at day 12 and increases in extent and intensity through day 15. Note that macrophages also exhibit strong anxA1 expression (red arrows). (E) shows gross appearance of lungs excised at the various time points. Note the progressively darker color as the B16-F10-Luc2 melanoma cells metastasize to the lungs. All scale bars = 10 μm.

B16-F10-Luc2 metastasis-bearing lungs were collected and assayed by IHC on day 0 (*n* = 3), day 9 (*n* = 3), day 12 (*n* = 3), and day 15 (*n* = 2) after IV cell implantation. On day 0, there was no evident metastases; the endothelial cells lining the lung microvessels were consistently negative for anxA1 (Fig 5A). On day 9, numerous small metastases were multifocally distributed throughout the pulmonary parenchyma. No obvious neovessels were apparent and no anxA1 staining was observed within the metastatic foci (Fig 5B). On day 12, numerous moderate-to-large, multifocal-to-coalescing foci of metastases were distributed throughout the pulmonary parenchyma and occupied approximately 50% of lung area. Neovessels were apparent within most metastatic foci, and one of the three animals exhibited 2–3+ staining in neovessels (Fig 5C). On day 15, approximately 60–70% of the pulmonary parenchyma was infiltrated and expanded by large, multifocal-to-coalescing foci of metastases. In two of the two animals, the neovessels of larger foci were lined by endothelial cells that exhibited 2–3+ staining of anxA1 (Fig 5D). Throughout the time course, endothelial cells in adjacent normal lung tissue remained almost completely negative for anxA1 staining, with only very rare positive staining observed.

## Discussion

Tumor vascular markers present a unique antitumor targeting opportunity due to their availability to circulating drug and potential to facilitate local accumulation of systemically administered antitumor agents [5, 6]. Previously published data on anxA1 expression in tumor vasculature has established the presence of a membrane-bound, N-terminal–truncated form of anxA1 on the luminal membrane of tumor-associated endothelium, and anxA1 has been proposed as a vascular antitumor target [19–21]. However, until now a systematic study of anxA1 expression in tumor and normal vasculature has not been published.

To characterize anxA1 expression in tumor vasculature, we assessed the binding characteristics of a large panel of antibodies to identify those that bind N-terminal–cleaved anxA1. A related family member protein anxA2 has a high degree of sequence homology to anxA1 (55%), is upregulated in cancer, and is known to be expressed on endothelium [62–64]; therefore binding to anxA2 was assessed to ensure antibody specificity. In addition, human-mouse cross-reactivity was sought to facilitate translational studies. Based on these criteria, no commercially available anti-anxA1 monoclonal antibodies tested demonstrated the required binding profile and specificity, underscoring the importance of properly characterizing anti-anxA1 antibodies used in IHC studies. Many published papers on anxA1 expression in cancer report the use of either polyclonal antibodies, which have high liability for cross-reaction with other annexins, or antibodies such as clone 29, which was shown in our study to recognize the cleavable anxA1 N-terminal domain.

Given the lack of suitable commercially available antibodies, we undertook a phage panning campaign to isolate antibodies that specifically bind anxA1 and detect anxA1_27-246_. From this campaign we isolated clone 4, an antibody which demonstrated specificity for anxA1 over other annexins as well as species cross-reactivity, sub-nanomolar affinity, and excellent specificity in staining cytosolic and membrane anxA1 in fixed IHC samples.

Using clone 4 to investigate vascular anxA1 expression in lung tumor, we observed that five of eight lung tumor samples stained positive for vascular anxA1 in 1–10% of total endothelial cells. In addition, 30 of 80 NSCLC TMA cores demonstrated positive staining for anxA1, whereas in contrast no vascular staining of anxA1 was observed in normal tissue samples with the exceptions of stomach fundus and kidney medulla cores. Tumor parenchyma staining was positive in a majority of NSCLC samples (54 of 80), which is consistent with previous reports of anxA1 expression in lung tumors [37–40]. Although induction of anxA1 expression in lung tumor vasculature was not as prevalent and widespread as that seen in other tumor vascular targets, notably B7-H3 [8] and PSMA [65, 66], these data validate anxA1 as a potential tumor vascular drug target in a proportion of human lung tumors.

To identify translatable rodent tumor models that could be used to study tumor vascular anxA1 expression, we also used IHC to assess vascular and neoplastic anxA1 expression in several rodent models of cancer. Because the induction of tumor endothelial markers involves a complex interplay between tumor parenchyma and stromal cells, we chose metastatic and orthotopic syngeneic models for this study, as these models more accurately reflect tumor growth within the tissue environment and do not involve compromised immune function [58, 60, 61]. In addition, it is believed that lung metastatic models replicate the mechanism of vascular recruitment seen in lung cancer [67, 68].

Two of the rodent models (B16-F10-Luc2 lung metastatic and Py230 mammary orthotopic) showed substantial induction of vascular anxA1 expression. In contrast to these models, the 4T1-Luc2, LLC-Luc2, and rat 13762 models were uniformly and consistently negative for vascular anxA1 expression, although each of these models exhibited anxA1 expression in tumor parenchymal cells to various degrees. In both the human lung tumor and the mouse models we tested, there was no apparent correlation between anxA1 expression levels in the tumor vasculature and the tumor parenchyma, suggesting that the function of anxA1 in these cell types may be independent of each other.

Previously, tumor uptake of anti-anxA1 antibody was observed in syngeneic rodent tumors generated by co-implantation of tumor spheroids with orthotopic tissue in a dorsal window chamber [19–21]. In the B16-F10-Luc2 lung metastatic model, which was shown by IHC to express anxA1 in the tumor neovasculature, anti-anxA1 antibodies 1 and 84 did not demonstrate significant tumor uptake upon systemic injection (S3 Fig). On the basis of these data, it appears that tumor uptake of anti-anxA1 antibodies may be dependent on the tumor model or involves the engagement of specific epitopes that are not addressed by these antibodies.

The defined time course of anxA1 expression in tumor vasculature in the B16-F10-Luc2 and Py230 models suggests a potential role for vascular anxA1 in facilitating or modulating growth in these tumors. Stromal-derived anxA1 has previously been shown to play a role in tumor angiogenesis, and anxA1 has been found to be induced in sprouting endothelial cells in an aortic ring assay [69]. In addition, it has been hypothesized that anxA1 could act to modulate the tumor immune environment, and anxA1 and its cleavage products are known to modulate neutrophil extravasation and transendothelial migration through interaction with formyl peptide receptors [52, 70, 71]. Additional studies are required to determine the functional role of vascular anxA1 in these tumor models.

In conclusion, we used phage panning techniques to isolate anti-anxA1 antibodies with binding profiles designed to target anxA1_27-346_, a truncated form of anxA1 that had previously been identified on the luminal surface of tumor endothelium. We used one such antibody for IHC studies to assess the prevalence and extent of vascular anxA1 expression in human NSCLC and normal tissues, as well as in a diverse set of rodent tumor models. Our results demonstrate that vascular anxA1 expression is present in a subset of human lung tumors and rodent tumor models, thus supporting its potential as a vascular tumor target for biologic agents. Studies of additional tumor indications and correlating rodent translational models will be useful for further assessment of anxA1 as a vascular tumor target.

## Supporting information

S1 Fig Binding characteristics of anti-anxA1 clones 1, 77, and 84. ELISA binding to recombinant mouse and human anxA1 and anxA1_27-346_. (A) EC_50_ values were as follows. Clone 1: mouse full-length anxA1, 3.75 nM; mouse anxA1_27-346_, 2.64 pM; human full-length anxA1, 1.98 pM; human anxA1_27-346_, 2.64 nM. Clone 77: mouse full-length anxA1, 9.88 pM; mouse anxA1_27-346_, 11.12 pM. Clone 84: mouse full-length anxA1, 27.93 pM; mouse anxA1_27-346_, 32.71 pM. (B) Antibody binding to cell-associated anxA1_27-346_ localized to the surface of anxA1-negative RAJI cells in the presence of 5 mM CaCl_2_, assessed by flow cytometry.
(TIF)

S2 Fig Representative Annexin A1 IHC images showing the variation in expression patterns seen in human NSCLC TMA samples. Left: A core showing expression in macrophages and neutrophils only with no endothelial (arrows) or neoplastic cell expression. Center: a core exhibiting positive neoplastic cell expression without endothelial cell expression. Right: A core exhibiting positive macrophage, neutrophil, and endothelial cell (arrows) expression but no neoplastic cell expression.
(TIF)

S3 Fig Biodistribution of anti-anxA1 antibodies. Shown are results at (A) 4 hours and (B) 24 hours. Alexa Fluor 680–labeled clones 1 and 84 and human IgG1 isotype control NIP228 were administered via IV injection to mice bearing B16-F10-Luc2 lung tumor metastases at 12 days after lung seeding of 0.5 × 10^6^ B16-F10-Luc2 cells via tail vein injection. No significant differences were observed between groups; *n* = 3 per group.
(TIF)

S1 Data (DOCX)
