# Correction: Upregulation of annexin A1 protein expression in the intratumoral vasculature of human non–small-cell lung carcinoma and rodent tumor models

**DOI:** 10.1371/journal.pone.0252060

**Published:** 2021-05-17

**Authors:** Kevin L. Allen, Jennifer Cann, Weiguang Zhao, Norman Peterson, Michelle Lazzaro, Haihong Zhong, Herren Wu, William F. Dall’Acqua, M. Jack Borrok, Melissa M. Damschroder, Ping Tsui, Qing Li

## Notice of republication

This article was republished on May 7, 2021 to correct an error in the Acknowledgments section. The corresponding author’s contact email was also updated. Please download this article again to view the correct version.
