# Supplementary figures and images for: Upregulation of annexin A1 protein expression in the intratumoral vasculature of human non–small-cell lung carcinoma and rodent tumor models
Source: PLoS One. 2020 Jun 4;15(6):e0234268. doi: 10.1371/journal.pone.0234268 (PMC7272081; doi:10.1371/journal.pone.0234268)

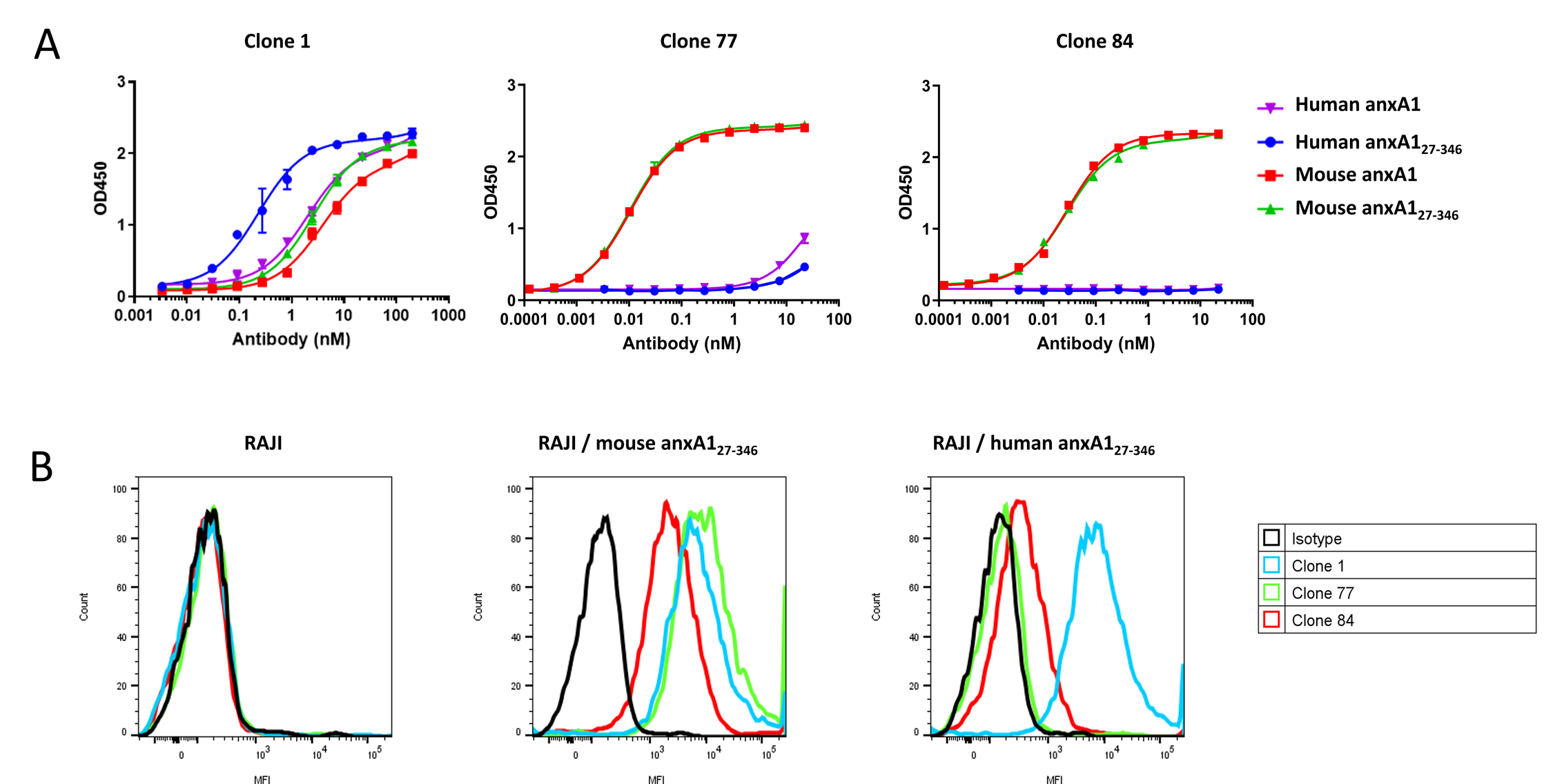

Supplement: S1 Fig — ELISA binding to recombinant mouse and human anxA1 and anxA127-346. (A) EC50 values were as follows. Clone 1: mouse full-length anxA1, 3.75 nM; mouse anxA127-346, 2.64 pM; human full-length anxA1, 1.98 pM; human anxA127-346, 2.64 nM. Clone 77: mouse full-length anxA1, 9.88 pM; mouse anxA127-346, 11.12 pM. Clone 84: mouse full-length anxA1, 27.93 pM; mouse anxA127-346, 32.71 pM. (B) Antibody binding to cell-associated anxA127-346 localized to the surface of anxA1-negative RAJI cells in the presence of 5 mM CaCl2, assessed by flow cytometry. (TIF) [file pone.0234268.s001.tif]

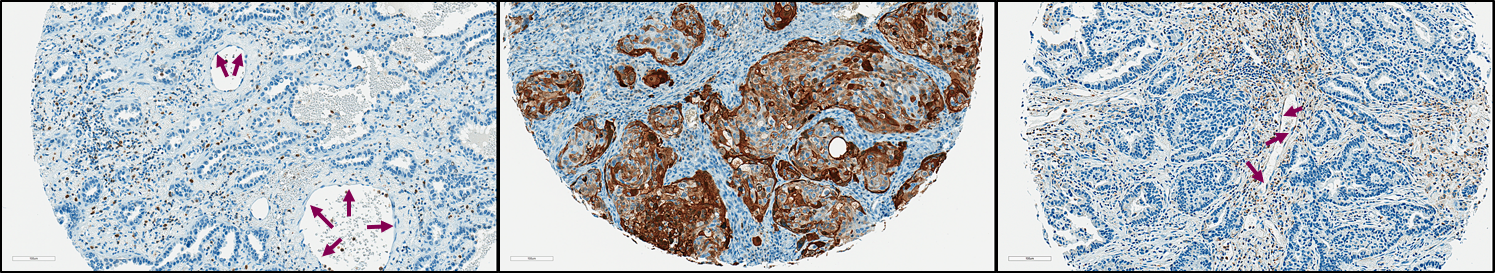

Supplement: S2 Fig — Left: A core showing expression in macrophages and neutrophils only with no endothelial (arrows) or neoplastic cell expression. Center: a core exhibiting positive neoplastic cell expression without endothelial cell expression. Right: A core exhibiting positive macrophage, neutrophil, and endothelial cell (arrows) expression but no neoplastic cell expression. (TIF) [file pone.0234268.s002.tif]

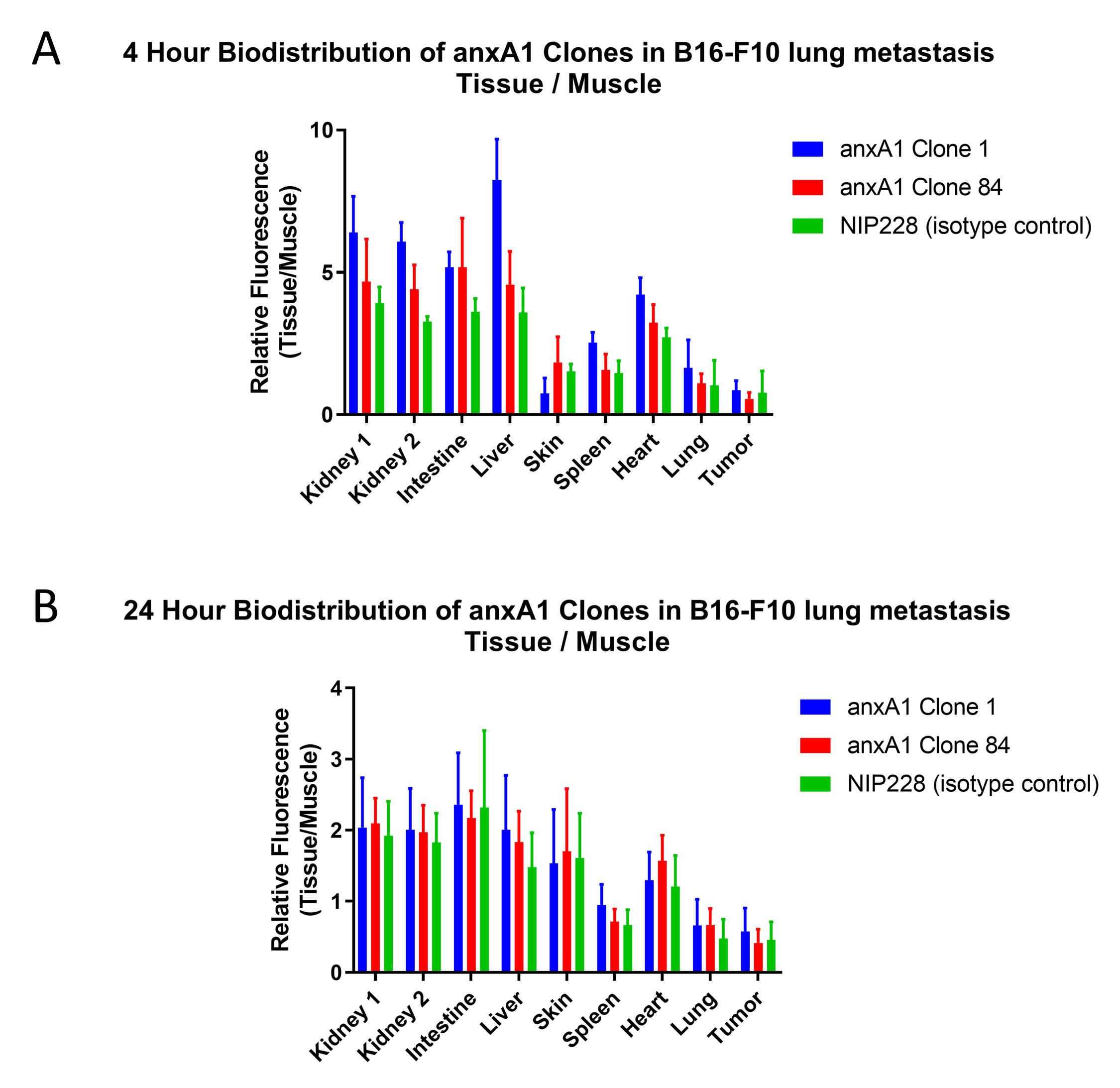

Supplement: S3 Fig — Shown are results at (A) 4 hours and (B) 24 hours. Alexa Fluor 680–labeled clones 1 and 84 and human IgG1 isotype control NIP228 were administered via IV injection to mice bearing B16-F10-Luc2 lung tumor metastases at 12 days after lung seeding of 0.5 × 10^6^ B16-F10-Luc2 cells via tail vein injection. No significant differences were observed between groups; n = 3 per group. (TIF) [file pone.0234268.s003.tif]
