# Supplementary material for: Upregulation of annexin A1 protein expression in the intratumoral vasculature of human non–small-cell lung carcinoma and rodent tumor models
Source: PLoS One. 2020 Jun 4;15(6):e0234268. doi: 10.1371/journal.pone.0234268 (PMC7272081; doi:10.1371/journal.pone.0234268)
Supplement: S1 Data — (DOCX) [file pone.0234268.s004.docx]

**Method**

**In vivo imaging system**

Anti-anxA1 antibodies and an isotype control antibody were labeled with Alexa Fluor 680 (SAIVI Rapid Antibody Labeling Kit; Life Technologies, Grand Island, NY) (degree of labeling, 2–3), and 2 mg/kg was injected IV into mice bearing B16-F10 lung metastases at 12 days after seeding. Major tissues were harvested from euthanized mice. Luminescence and surface fluorescence from the tissues were sequentially analyzed with a Spectrum in vivo imaging system (PerkinElmer, Waltham, MA) set at medium binning, F-stop 1, and auto exposure. Luminescence and surface fluorescence of the tissues were quantitated as average radiance (p/s/cm^2^/sr) by using Living Image software (PerkinElmer). Luminescence and surface fluorescence signals were also standardized across mice and presented as a ratio of the signal from skeletal muscle. Data were analyzed with Prism software, version 8 (GraphPad, La Jolla, CA). Analysis of variance and Bonferroni post hoc analyses were performed to determine statistical significance (defined as *P* < 0.05).
